# Integrating Bacteriophage onto the Magnetic Nanozyme for Effective Enrichment and Colorimetric Detection of *Cronobacter sakazakii* in Powdered Infant Formula

**DOI:** 10.3390/foods13233788

**Published:** 2024-11-25

**Authors:** Xuechao Xu, Yuansong Zhang, Lu Gao, Juanli Yang, Zhenquan Yang

**Affiliations:** School of Food Science and Engineering, Yangzhou University, Yangzhou 225127, China; xuechaoxu@yzu.edu.cn (X.X.); yuansongzhang_yzu@126.com (Y.Z.); gaolu@yzu.edu.cn (L.G.); y1948327385@163.com (J.Y.)

**Keywords:** *Cronobacter sakazakii*, bacteriophage EspYZU13, magnetic nanozyme, bacterial enrichment, colorimetric detection, powdered infant formula

## Abstract

*Cronobacter sakazakii* is a dangerous pathogen easily found in powdered infant formula (PIF), causing severe infections and even death in infants. Herein, a bacteriophage-immobilized magnetic nanozyme (Fe_3_O_4_@EspYZU13) was prepared for *C. sakazakii* detection. Bacteriophage EspYZU13 isolated and identified by our group exhibits specific lytic capacity. Fe_3_O_4_@EspYZU13 possesses remarkable enrichment capacity towards *C. sakazakii*, efficiently enriching different concentrations of *C. sakazakii* from a mixed bacterial solution. Furthermore, Fe_3_O_4_@EspYZU13 shows peroxidase-like activity, which can catalyze the 3,3′,5,5′-tetramethylbenzidine (TMB) chromogenic reaction in the presence of H_2_O_2_. Upon introduction of *C. sakazakii*, it can be specifically captured by Fe_3_O_4_@EspYZU13, inhibiting its peroxidase-like activity. Based on it, *C. sakazakii* ranging from 3.2 × 10^1^ to 3.2 × 10^7^ CFU mL^−1^ can be determined, offering a detection limit (LOD) of 26 CFU mL^−1^. Moreover, this reaction system keeps high specificity towards *C. sakazakii*, which can resist interferences of other possible coexisting bacteria. *C. sakazakii* in the artificially contaminated PIF can be detected, offering good recoveries (96.76% to 103.13%). These results indicate that our proposed reaction system demonstrates its practical application potential for efficient enrichment of *C. sakazakii* from complex samples and accurate determination of *C. sakazakii* in PIF.

## 1. Introduction

*Cronobacter sakazakii* (*C. sakazakii*), frequently identified in powdered infant formula (PIF), is an opportunistic pathogen linked to foodborne illnesses [1,2]. The contamination of PIF with *C. sakazakii* can potentially occur at various stages, encompassing manufacturing, processing, and transportation [3]. Ingestion of *C. sakazakii*-contaminated PIF by infants can result in severe infections, such as meningitis, bacteremia, and even death. Currently, available data indicate that mortality rates for infants consuming *C. sakazakii*-contaminated PIF range from 40% to 80% [3]. Since 1958, over a hundred cases of infections caused by *C. sakazakii* have been reported worldwide. Therefore, it is imperative to develop a specific and sensitive assessment technique for the detection of *C. sakazakii* in PIF.

Nowadays, determination of *C. sakazakii* primarily relies on the conventional microbial culture method [4]. Although this approach enables precise detection outcomes, it necessitates proficient operators and entails an extended culture duration (approximately 5 days), thereby significantly impeding further practical implementation. To overcome these limitations, researchers are currently engaged in the development of innovative and accelerated detection methodologies based on molecular biology, immunology, and so on [5,6]. For instance, Hu et al. proposed a rapid detection method for *C. sakazakii* by real-time PCR, achieving a detection limit (LOD) of 110 CFU mL^−1^ [7]. Chen et al. reported the utilization of microfluidic chips combined with isothermal amplification analysis for detecting *C. sakazakii* in PIF, offering a LOD of 2.2 × 10^3^ CFU g^−1^ [8]. These methods are also limited by laborious detection procedures, expensive testing reagents, and poor detection sensitivity. Given these constraints, there is an urgent imperative to develop a pioneering detection methodology that surpasses these drawbacks and offers a more streamlined and dependable approach for determining *C. sakazakii* in PIF.

Nanozymes, also referred to as nanoscale enzyme mimetics, represent an intriguing category of nanomaterials that exhibit exceptional natural enzyme-like properties by seamlessly integrating the catalytic functionality of natural enzymes with the distinctive physicochemical characteristics inherent in nanomaterials [9,10]. The exceptional stability and enhanced catalytic activity of nanozymes surpass those of natural enzymes, establishing a solid foundation for their extensive application in biochemical analysis, particularly in bacterial detection [11,12]. For example, Yu’s group proposed a dual-function magnetic nanozyme for bacteria detection with a low LOD of 26 CFU mL^−1^ [13]. A significant drawback of nanozyme-based bacteria detection lies in their limited specificity towards target analytes. To address this issue, numerous specific recognition units, such as functional groups, aptamers, and antibodies, have been immobilized on the surface of nanozymes to enhance their specificity towards target bacteria [14,15]. For instance, Xia’s group prepared an electrochemical detector based on a functional two-dimensional metal–organic framework nanozyme for the specific detection of *Staphylococcus aureus* (*S. aureus*) via an anti-*S. aureus* antibody, achieving a low LOD of 6 CFU mL^−1^ [16]. However, these specific recognition units are still limited in terms of their limited recognition ability and high cost. Therefore, it is crucial to explore alternative specific recognition units that can overcome these drawbacks.

Bacteriophages, also known as phages, refer to a type of virus that specifically infects and replicates within bacteria [17]. Bacteriophages can be used not only to directly combat infections but also as tools for monitoring bacteria. For instance, Zhou et al. used a T2 bacteriophage modified nanostructured electrode to realize electrochemical detection of *Escherichia coli* (*E. coli*) with high specificity and sensitivity [18]. These remarkable outcomes observed can be attributed to the following significant advantages of bacteriophages: (1) bacteriophages exhibit exceptional specificity towards diverse bacterial strains; (2) the abundance and diversity of bacteriophages in natural environments provide a vast reservoir for discovering novel phage species with distinctive properties [19,20]. Inspired by these advantages of nanozymes and bacteriophages, a combination of nanozymes and bacteriophages is anticipated to achieve the highly efficient detection of *C. sakazakii* in PIF.

**Scheme 1:**
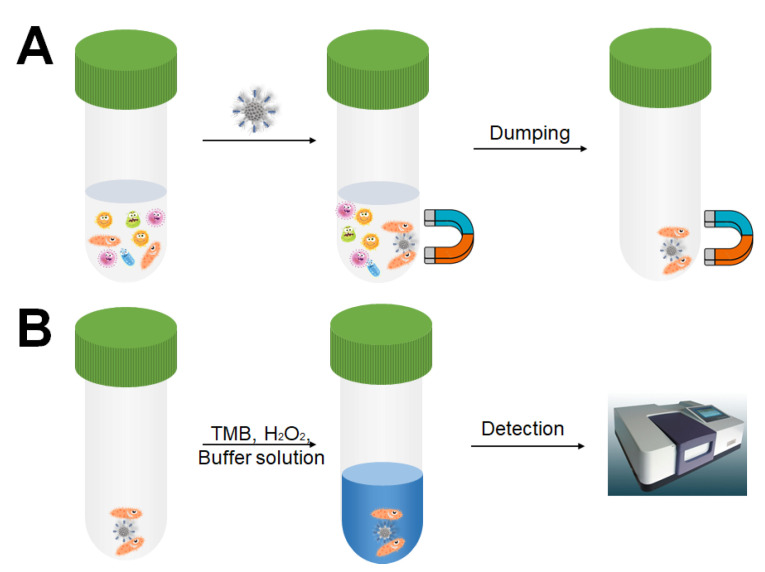
Effective enrichment (**A**) and colorimetric detection procedure (**B**) of *C. sakazakii* using Fe_3_O_4_@EspYZU13.

The present study proposes the utilization of a bacteriophage-immobilized nanozyme for the highly efficient detection of *C. sakazakii* in PIF (Scheme 1). Initially, our group isolated and identified the bacteriophage EspYZU13, which can effectively and specifically lyse *C. sakazakii*. Subsequently, bacteriophage EspYZU13 was immobilized onto the magnetic nanozyme Fe_3_O_4_, preparing Fe_3_O_4_@EspYZU13. Remarkably, Fe_3_O_4_@EspYZU13 exhibits exceptional peroxidase-like activity capable of catalyzing the 3,3′,5,5′-tetramethylbenzidine (TMB) chromogenic reaction. Consequently, specific inhibition of peroxidase-like activity of Fe_3_O_4_@EspYZU13 by *C. sakazakii* leads to specific colorimetric detection of *C. sakazakii*. Furthermore, Fe_3_O_4_@EspYZU13 demonstrates excellent magnetic enrichment capacity, enabling convenient enrichment of *C. sakazakii* from complex samples. This magnetic enrichment capability of Fe_3_O_4_@EspYZU13 enables rapid concentration of the target analytes, thereby enhancing the sensitivity of detection. Considering this, Fe_3_O_4_@EspYZU13 can be utilized for the colorimetric detection of *C. sakazakii* in PIF with exceptional specificity and sensitivity.

## 2. Materials and Methods

### 2.1. Chemicals and Reagents

The detailed chemicals and reagents are displayed in SM (Supplementary Materials).

### 2.2. Bacteria and Bacteriophage Culture

Herein, the following bacterial strains, which were stored in our lab, were utilized: *C. sakazakii* (CICC 21569), *E. coli* (CICC 10664), *S. aureus* (CICC 21600), *Bacillus cereus* (*B. cereus*, CICC 21261), *Listeria monocytogenes* (*L. monocytogenes*, ATCC 19111), *Salmonella enteritidis* (*S. enteritidis*, CICC 21513), and *Enterobacter hormaechei* (*E. hormaechei*, Eh-YZU05). All bacteria were cultured overnight under incubation conditions of 37 °C and a speed of 120 rpm. *C. sakazakii* was cultivated in nutrient broth (NB) medium, while the remaining bacteria were grown in Luria–Bertani (LB) medium [21].

Bacteriophage EspYZU13 was cultured as follows: 200 μL purified bacteriophage EspYZU13 and 200 μL host bacterium *C. sakazakii* were combined, followed by the addition of 10 mL LB culture medium. The mixture was then incubated at 37 °C for 8 h. Subsequently, the mixture was centrifuged at 8000 rpm for 1 min and filtered using a sterile membrane with a pore size of 0.22 nm. The resulting filtrate was stored at 4 °C for future use [21].

### 2.3. Synthesis of Fe_3_O_4_@EspYZU13

Magnetic nanozyme Fe_3_O_4_ was accomplished using a one-pot method according to previously reported work [22]. In brief, FeCl_3_·6H_2_O (4.4 g) and FeCl_2_·4H_2_O (1.7 g) were dispersed in 80 mL deionized water and incubated at 70 °C under magnetic agitation for 30 min. Subsequently, NH_4_OH solution (25~28%) (10 mL) was added into the above mixture under continuous magnetic agitation at 70 °C for 30 min. Then, ascorbic acid solution (0.085 mol L^−1^, 4 mL) was slowly introduced, incubating at 90 °C under magnetic agitation for 60 min. After cooling to room temperature, the resultant product was subjected to magnetic separation and subsequently washed multiple times with deionized water.

Fe_3_O_4_@EspYZU13 was synthesized through bonding between Fe_3_O_4_ and EspYZU13 [23]. Specifically, 10 mg Fe_3_O_4_ was added to a 1 mL aqueous solution containing 1-(3-dimethylaminopropyl)-3-ethylcarbodiimide hydrochloride (EDC·HCl)/N-hydroxy succinimide (NHS) (20 mg mL^−1^ EDC HCl and 20 mg mL^−1^ NHS) and 4-morpholineethanesulfonic acid (MES, 10 mmol L^−1^, pH 6.0). The mixture was vortexed and activated at room temperature for 30 min. Subsequently, 1.5 mL bacteriophage EspYZU13 (10^8^ PFU mL^−1^) was introduced into the Fe_3_O_4_ solution, followed by incubation at 37 °C for 12 h. The obtained Fe_3_O_4_@EspYZU13 was collected by magnetic separation and then dispersed in 10 mL deionized water. The resultant Fe_3_O_4_@EspYZU13 mother solution was stored at 4 °C for further utilization. According to inductively coupled plasma optical emission spectrometer (ICP-OES) analysis, the Fe element in the EspYZU13@Fe_3_O_4_ mother solution was detected as approximately 1.38 mg g^−1^.

### 2.4. Characterization

The samples were characterized using the following equipment. Transmission electron microscope (TEM) images were acquired from a Tecnai 12 (Philips, Amsterdam, The Netherlands) at a voltage of 100 kV. X-ray diffraction (XRD) measurements: X-ray diffraction (XRD) analysis was conducted utilizing an X-ray diffractometer, D8 Advance (Bruker AXS, Billerica, MA, USA). Laser scanning confocal microscopy (LSCM) images was obtained from a laser scanning confocal microscope, LSM 880NLO (Carl Zeiss, Oberkochen, Germany). Free radicals were assessed utilizing an electron paramagnetic resonance spectrometer (EPR, A300-10/12, Bruker, Billerica, MA, USA). The Fe element in the mother solution EspYZU13@Fe_3_O_4_ was determined using an inductively coupled plasma optical emission spectrometer (ICP-OES), Perkin Elmer Optima 8000, Waltham, MA, USA. Absorbance measurements were performed using a UV-Vis spectrophotometer, UV-3200, Mapada, Shanghai, China.

### 2.5. Magnetic Enrichment of C. sakazakii Using Fe_3_O_4_@EspYZU13

Magnetic enrichment of *C. sakazakii* from a pure bacterial solution: 100 μL *C. sakazakii* (10^3^ and 10^7^ CFU mL^−1^) and 100 μL Fe_3_O_4_@EspYZU13 were mixed and incubated for 13 min, followed by magnetic separation. Subsequently, the supernatant liquid was diluted with a gradient of 0.9% saline solution. Appropriate dilutions were spread onto NB solid plates, then incubated at 37 °C for 18 h.

Magnetic enrichment of *C. sakazakii* from a mixed bacterial solution: the bacterial strains, including 1 mL *S enteritidis* (10^3^ and 10^7^ CFU mL^−1^), 1 mL *S. aureus* (10^3^ and 10^7^ CFU mL^−1^, 1 mL *B. cereus* (10^3^ and 10^7^ CFU mL^−1^), 1 mL *L. monocytogenes* (10^3^ and 10^7^ CFU mL^−1^), 1 mL *E. hormaechei* (10^3^ and 10^7^ CFU mL^−1^), and 1 mL *C. sakazakii* (10^3^ and 10^7^ CFU mL^−1^) were condensed to 1 mL mixed bacterial solution by centrifugation. A quantity of 100 μL mixed bacterial solution (10^3^ and 10^7^ CFU mL^−1^) and 100 μL Fe_3_O_4_@EspYZU13 was mixed and incubated for 13 min, followed by magnetic separation. Subsequently, the supernatant liquid was diluted with a gradient of 0.9% saline solution. Appropriate dilutions were spread onto *C. sakazakii* chromogenic medium (DFI agar), then incubated at 37 °C for 18 h.

### 2.6. Peroxidase-Mimic Activity of Fe_3_O_4_@EspYZU13

A quantity of 100 μL Fe_3_O_4_@EspYZU13 and 100 μL H_2_O_2_ (100 mM), along with 100 μL TMB (5 mM, dissolved in ethanol), was added into 2700 μL NaAc-HAc buffer solution (0.2 M, pH 4.0) in turn and then incubated for 19 min. Subsequently, the absorbance (500 nm~800 nm) of the mixture was promptly analyzed using a UV-Vis spectrophotometer.

### 2.7. Colorimetric Detection of C. sakazakii Using Fe_3_O_4_@EspYZU13

A quantity of 100 μL *C. sakazakii* solution (ranging from 9.6 × 10^2^ CFU mL^−1^ to 9.6 × 10^8^ CFU mL^−1^) and 100 μL Fe_3_O_4_@EspYZU13 was mixed, incubated for 13 min. After that, 2600 μL NaAc-HAc buffer solution (0.2 M, pH 4.0), 100 μL H_2_O_2_ (100 mM), and 100 μL TMB (5 mM, dissolved in ethanol) were added by turns. The resultant mixture was further incubated for 19 min before analysis using a UV-Vis spectrophotometer ranging from 500 nm to 800 nm.

### 2.8. Actual Food Sample Detection

Preparation of artificial *C. sakazakii*-contaminated PIF samples: 900 μL PIF solution and 100 μL *C. sakazakii* were evenly mixed.

Detection of *C. sakazakii* in artificially contaminated PIF samples: 100 μL artificially contaminated PIF solution and 100 μL Fe_3_O_4_@EspYZU13 were incubated for 13 min. Then, 100 μL H_2_O_2_ (100 mM), 100 μL TMB (5 mM, dissolved in ethanol), and 2600 μL NaAc-HAc buffer solution (0.2 M, pH 4.0) were added. The mixture was further incubated for 19 min before analysis using a UV-Vis spectrophotometer.

### 2.9. Data Processing

The data in this study were processed using Origin 2022 software (OriginLab Corporation, Northampton, MA, USA) and Excel software (https://www.microsoft.com/en-us/microsoft-365/excel).

## 3. Results and Discussion

### 3.1. Synthesis and Characterization

**Figure 1:**
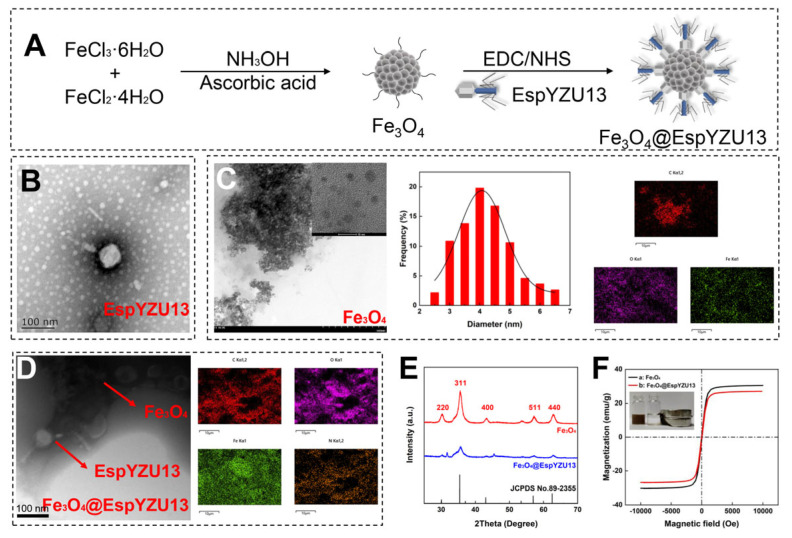
Preparation of Fe_3_O_4_@EspYZU13 (**A**), TEM image of EspYZU13 (**B**), TEM images and element mapping of Fe_3_O_4_ (**C**) and Fe_3_O_4_@EspYZU13 (**D**), and XRD spectra (**E**) and hysteresis loops (**F**) of Fe_3_O_4_ and Fe_3_O_4_@EspYZU13.

Fe_3_O_4_@EspYZU13 was synthesized via immobilization of EspYZU13 on the surface of Fe_3_O_4_ (Figure 1A). At first, bacteriophage EspYZU13 was isolated by our research team [24]. According to the Ninth Report of the International Committee on Virus Taxonomy, EspYZU13 is classified as a member of the *Myoviridae* family [25]. As depicted in Figure 1B, the obtained EspYZU13 exhibits a regular polyhedron-shaped head with a diameter of approximately 74 nm. It possesses a characteristic freely contractile tail sheath measuring around 102 nm in length. According to previous research, EspYZU13 exhibits specific lytic capacity towards its host bacterium *C. sakazakii* [24].

Then, TEM imaging of Fe_3_O_4_ (Figure 1C) reveals its interconnected formation. High-resolution TEM analysis demonstrates a uniform distribution of Fe_3_O_4_, with a diameter ranging from approximately 2.5 nm to 6.5 nm and an average diameter of about 4.1 nm. Furthermore, element mapping confirms homogeneous distributions of carbon (C), oxygen (O), and iron (Fe) elements within the Fe_3_O_4_ structure. Additionally, XRD characterization shows five distinct characteristic peaks at 30.3°, 35.5°, 43.4°, 57.2°, and 62.8°, corresponding to crystal planes (220), (311), (400), (511), and (440) of Fe_3_O_4_ (JCPDS No.89-2355) (Figure 1E). Moreover, hysteresis loop analysis demonstrates its exceptional magnetic property (Figure 1F). These findings collectively affirm the successful synthesis of Fe_3_O_4_.

Finally, TEM imaging clearly reveals the tight binding between Fe_3_O_4_ and EspYZU13 (Figure 1D) after immobilizing EspYZU13 onto the Fe_3_O_4_. Furthermore, XRD analysis demonstrates that the structure of Fe_3_O_4_@EspYZU13 remains unchanged after covalent binding to EspYZU13, as evidenced by its consistency with that of pure Fe_3_O_4_. In addition, the diminished peak intensity of XRD suggests that the surface of Fe_3_O_4_ is enveloped by EspYZU13. These results indicate the successful preparation of Fe_3_O_4_@EspYZU13. Moreover, an hysteresis loop of Fe_3_O_4_@EspYZU13 was recorded (Figure 1F), revealing its exceptional magnetic properties after covalent immobilization of EspYZU13 and highlighting its potential for magnetic enrichment towards *C. sakazakii*.

### 3.2. Enrichment of C. sakazakii Using Fe_3_O_4_@EspYZU13

**Figure 2:**
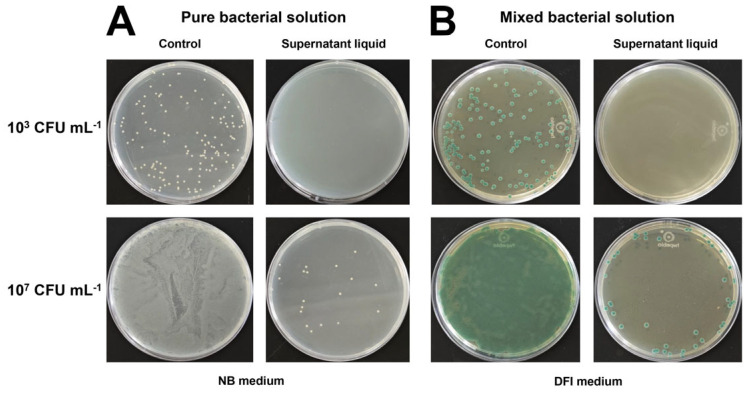
Enrichment of *C. sakazakii* (10^3^ and 10^7^ CFU mL^−1^) using Fe_3_O_4_@EspYZU13 from the pure bacterial solution (**A**) and the mixed bacterial solution (*S enteritidis*, *S. aureus*, *B. cereus*, *L. monocytogenes*, *E. hormaechei*, and *C. sakazakii*) (**B**).

In view of the magnetic performance and specific capture ability of Fe_3_O_4_@EspYZU13, it was utilized for rapid enrichment of *C. sakazakii*. At first, Fe_3_O_4_@EspYZU13 was employed to enrich *C. sakazakii* from a pure bacterial solution. As shown in Figure 2A, both 10^3^ and 10^7^ CFU mL^−1^ *C. sakazakii* can be well enriched. The supernatant liquid of 10^3^ CFU mL^−1^ *C. sakazakii* displayed undetectable levels. Moreover, the supernatant liquid of 10^7^ CFU mL^−1^ *C. sakazakii* demonstrated only a few of colonies observed in the NB medium. Moreover, Fe_3_O_4_@EspYZU13 was utilized for rapid enrichment of 10^3^ and 10^7^ CFU mL^−1^ *C. sakazakii* from a mixed bacterial solution. Residual bacteria in the supernatant liquid were detected using *C. sakazakii* chromogenic medium (DFI medium). As shown in Figure 2B, no blue-green colonies were observed on the DFI medium from the supernatant liquid of a mixed bacterial solution (10^3^ CFU mL^−1^), while only dozens of blue-green colonies were observed on the DFI medium from the supernatant liquid of a mixed bacterial solution (10^7^ CFU mL^−1^). These findings underscore the robust enrichment performance exhibited by Fe_3_O_4_@EspYZU13 towards *C. sakazakii*.

### 3.3. Peroxidase-Mimicking Activity of Fe_3_O_4_@EspYZU13

**Figure 3:**
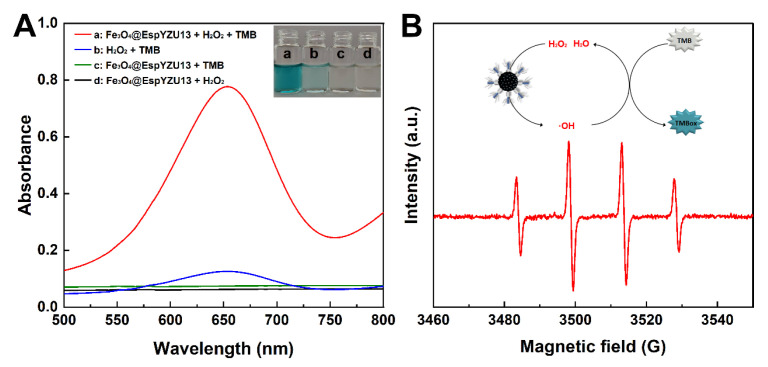
Absorbance of Fe_3_O_4_@EspYZU13 + H_2_O_2_ + TMB, H_2_O_2_ + TMB, Fe_3_O_4_@EspYZU13 + TMB, and Fe_3_O_4_@EspYZU13 + H_2_O_2_ reaction systems (inset is the corresponding real image) (**A**), and EPR spectrum of the Fe_3_O_4_@EspYZU13 + H_2_O_2_ + DMPO system (inset is the inherent mechanism of TMB chromogenic reaction catalyzed by peroxidase-like Fe_3_O_4_@EspYZU13 in presence of H_2_O_2_) (**B**).

The absorbance of Fe_3_O_4_@EspYZU13 + H_2_O_2_ + TMB, H_2_O_2_ + TMB, Fe_3_O_4_@EspYZU13 + TMB, and Fe_3_O_4_@EspYZU13 + H_2_O_2_ reaction systems was recorded, as depicted in Figure 3A. Notably, the Fe_3_O_4_@EspYZU13 + H_2_O_2_ + TMB reaction system exhibited a prominent absorption peak at 652 nm, indicating the formation of blue oxidized TMB (TMBox). Conversely, both the other reaction systems (Fe_3_O_4_@EspYZU13 + TMB and Fe_3_O_4_@EspYZU13 + H_2_O_2_) did not exhibit any significant absorption peak at 652 nm. And the H_2_O_2_ + TMB reaction system only showed a little absorption peak at 652 nm. These findings suggest that Fe_3_O_4_@EspYZU13 can effectively accelerate the TMB chromogenic reaction. To elucidate the underlying mechanism of Fe_3_O_4_@EspYZU13 + H_2_O_2_ + TMB, the generation of free radicals was confirmed through EPR analysis using 5, 5-dimethyl-1-pyrroline N-oxide (DMPO) as a capture agent. As depicted in Figure 3B, the resulting curve exhibits four peaks that align with the characteristic peaks of hydroxyl radical (·OH). Consequently, it can be inferred that Fe_3_O_4_@EspYZU13 possesses peroxidase-like activity capable of catalyzing the conversion of H_2_O_2_ into hydroxyl radicals, thereby oxidizing colorless TMB to yield blue TMBox (Figure 3B inset).

In addition, effect of reaction time, buffer solution pH, and storage time on the Fe_3_O_4_@EspYZU13 + H_2_O_2_ + TMB reaction system were investigated. At first, the time-dependent absorbance change at 652 nm of four reaction systems was recorded. As depicted in Figure S1, the Fe_3_O_4_@EspYZU13 + H_2_O_2_ + TMB reaction system exhibited a gradual increase in absorbance at 652 nm, ultimately reaching its maximum at approximately 19 min. Therefore, based on these findings, the optimal reaction time was determined as 19 min. Moreover, the peroxidase-like activity of Fe_3_O_4_@EspYZU13 is significantly influenced by buffer solution pH. As illustrated in Figure S2, Fe_3_O_4_@EspYZU13 exhibits its highest peroxidase-like enzyme activity at pH 4.0, thereby establishing it as the optimal reaction condition for this experimental study. Then, the storage stability of Fe_3_O_4_@EspYZU13 was also assessed. As depicted in Figure S3, the peroxidase-like activity of Fe_3_O_4_@EspYZU13 remained virtually unchanged over a period of 30 days, thus demonstrating its excellent storage stability.

Additionally, steady-state kinetics of Fe_3_O_4_@EspYZU13 were investigated at the optimal conditions. The obtained data, as illustrated in Figures S4 and S5, demonstrate conformity with the Michaelis–Menten equation. Specifically, the *K*_m_ values were determined to be 1.4 mM for H_2_O_2_ and 0.17 mM for TMB, respectively, while the corresponding *V*_max_ values were found to be 1.2 × 10^−8^ M s^−1^ for H_2_O_2_ and 3.3 × 10^−8^ M s^−1^ for TMB, respectively. Notably, as listed in Table S1, Fe_3_O_4_@EspYZU13 exhibits enzyme-like catalytic performance comparable to other previously reported peroxidase-like nanozymes [21,26], thereby significantly enhancing its potential application in the detection and analysis of foodborne pathogens.

### 3.4. Colorimetric Detection of C. sakazakii Using Fe_3_O_4_@EspYZU13

**Figure 4:**
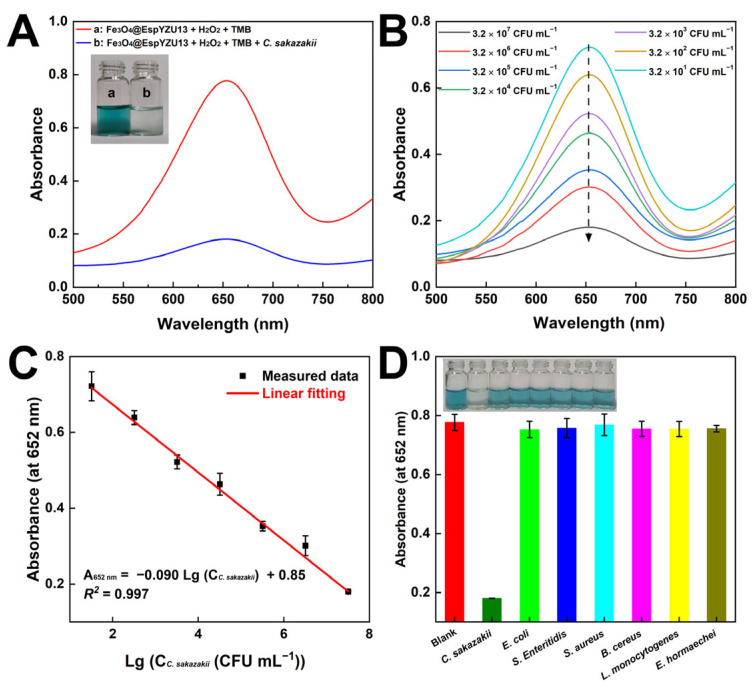
UV-Vis spectra of Fe_3_O_4_@EspYZU13 + H_2_O_2_ + TMB reaction system with/without *C. sakazakii* (**A**), UV-Vis spectra of Fe_3_O_4_@EspYZU13 + H_2_O_2_ + TMB reaction system with different concentrations of *C. sakazakii* (**B**), linear fitting between absorbance (652 nm) and logarithm of *C. sakazakii* concentration (**C**), and specificity of the Fe_3_O_4_@EspYZU13 + H_2_O_2_ + TMB reaction system towards *C. sakazakii* (**D**).

Given the excellent peroxidase-like activity of Fe_3_O_4_@EspYZU13, it was utilized for the detection of *C. sakazakii* in PIF. As depicted in Figure 4A, upon introduction of *C. sakazakii* (9.6 × 10^8^ CFU mL^−1^), the absorbance at 652 nm of the Fe_3_O_4_@EspYZU13 + H_2_O_2_ + TMB reaction system significantly decreases. This observation suggests that *C. sakazakii* can inhibit the peroxidase-like activity and impede the TMB chromogenic reaction. Furthermore, we also investigated the effects of both live and dead *C. sakazakii* (9.6 × 10^8^ CFU mL^−1^) on the Fe_3_O_4_@EspYZU13 + H_2_O_2_ + TMB reaction system (Figure S6). Compared to inhibitory effect of live *C. sakazakii* on this system, that exerted by dead *C. sakazakii* is negligible. Therefore, it can be concluded that only live *C. sakazakii* can hinder the TMB chromogenic system catalyzed by the peroxidase-like Fe_3_O_4_@EspYZU13, which profits from the specific recognition ability of Fe_3_O_4_@EspYZU13 towards live *C. sakazakii*.

**Figure 5:**
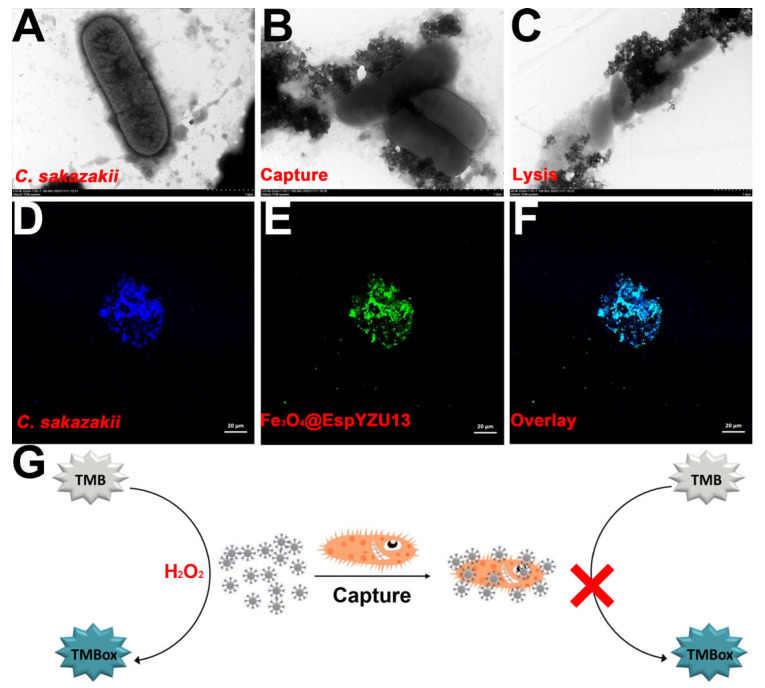
TEM images of *C. sakazakii* (**A**), *C. sakazakii* captured by Fe_3_O_4_@EspYZU13 (**B**), *C. sakazakii* lysed by Fe_3_O_4_@EspYZU13 (**C**), LSCM images of *C. sakazakii* (**D**), Fe_3_O_4_@EspYZU13 (**E**), overlay of *C. sakazakii* and Fe_3_O_4_@EspYZU13 (**F**), and the detection mechanism of the Fe_3_O_4_@EspYZU13 + H_2_O_2_ + TMB reaction system towards *C. sakazakii* (**G**).

In consideration of the inhibitory effect of *C. sakazakii* on the Fe_3_O_4_@EspYZU13 + H_2_O_2_ + TMB reaction system, various concentrations of *C. sakazakii* were introduced. As depicted in Figure 4B, upon addition of different concentrations of *C. sakazakii*, a gradual decrease in the absorption peak at 652 nm of the Fe_3_O_4_@EspYZU13 + H_2_O_2_ + TMB reaction system was observed (Figure 4B). As shown in Figure 4C, the absorbance at 652 nm of the Fe_3_O_4_@EspYZU13 + H_2_O_2_ + TMB reaction system was linearly correlated with the logarithm of *C. sakazakii* concentration, yielding a linear equation, A_652 nm_= −0.090 Lg (C*_C. sakazakii_*) + 0.85, with a LOD calculated as low as 26 CFU mL^−1^. The specificity of the Fe_3_O_4_@EspYZU13 + H_2_O_2_ + TMB reaction system towards *C. sakazakii* was further investigated (Figure 5D). Minimal interference from other possible coexisting bacteria (including *E. coli* (9.6 × 10^8^ CFU mL^−1^), *S. enteritidis* (9.6 × 10^8^ CFU mL^−1^), *S. aureus* (9.6 × 10^8^ CFU mL^−1^), *B. cereus* (9.6 × 10^8^ CFU mL^−1^), *L. monocytogenes* (9.6 × 10^8^ CFU mL^−1^), and *E. hormaechei* (9.6 × 10^8^ CFU mL^−1^)) on the chromogenic system was observed, highlighting its exceptional specificity towards *C. sakazakii*. Compared with previously reported methods (Table S2), our proposed Fe_3_O_4_@EspYZU13 + H_2_O_2_ + TMB reaction system exhibits the widest detection range towards *C. sakazakii* [5,27,28,29]. Furthermore, the remarkably low LOD of this reaction system suggests its potential for accurately determining ultralow concentrations of *C. sakazakii*-contaminated PIF.

### 3.5. Sensing Mechanisms of C. sakazakii Using Fe_3_O_4_@EspYZU13

To further investigate the inherent detection mechanism of the Fe_3_O_4_@EspYZU13 + H_2_O_2_ + TMB reaction system towards *C. sakazakii*, TEM was further employed to characterize the compounds of Fe_3_O_4_@EspYZU13 and *C. sakazakii*. As shown in Figure 5A, *C. sakazakii* retains its complete biological structure. Upon addition of Fe_3_O_4_@EspYZU13, *C. sakazakii* can be rapidly captured due to the specific recognition ability of EspYZU13. As shown in Figure 5B, Fe_3_O_4_@EspYZU13 and *C. sakazakii* are tightly bound together. Over time, Fe_3_O_4_@EspYZU13 effectively induces lysis of *C. sakazakii*, leading to the release of bacterial contents (Figure 5C). The confirmation of this phenomenon was once again validated by LSCM. As illustrated in Figure 5D, *C. sakazakii* was stained with 4′,6-diamidino-2-phenylindole (DAPI), exhibiting a blue fluorescence signal. Additionally, Fe_3_O_4_@EspYZU13 was labeled with SYBR-Gold nucleic acid gel stain (SYBR), displaying a green fluorescence signal (Figure 5E). Furthermore, as depicted in Figure 5F, the overlapping of both blue and green fluorescence signals indicates the specific capture of *C. sakazakii* by Fe_3_O_4_@EspYZU13. According to the whole genome sequencing and bioinformatics analysis, EspYZU13 harbors genes encoding tail proteins (ORF03, 40, and 43). EspYZU13 can specifically recognize and capture *C. sakazakii* through receptor-binding proteins, thereby obstructing the catalytic sites of Fe_3_O_4_@EspYZU13 [24]. This leads to a reduction in the peroxidase-like activity of Fe_3_O_4_@EspYZU13 and hampers the TMB chromogenic reaction catalyzed by Fe_3_O_4_@EspYZU13 in presence of H_2_O_2_ (Figure 5G).

### 3.6. Actual Food Sample Detection

To validate the detection performance of the Fe_3_O_4_@EspYZU13 + H_2_O_2_ + TMB reaction system in practical applications, we investigate its efficacy in detecting *C. sakazakii* in artificially contaminated PIF. As depicted in Table 1, the reaction system demonstrates exceptional detection capabilities for artificially *C. sakazakii*-contaminated PIF samples, exhibiting detection recoveries ranging from 96.76% to 103.13% and a relative standard deviation below 4.21%. Moreover, the obtained results align with those acquired through the traditional PC method, affirming the reliability of our established reaction system.

## 4. Conclusions

In this work, a bacteriophage was integrated onto the magnetic nanozyme to from Fe_3_O_4_@EspYZU13. Fe_3_O_4_@EspYZU13 exhibits the following characteristics: (1) a strong and rapid enrichment ability towards *C. sakazakii* from complex samples, which can enhance detection sensitivity and minimize interference from other coexisting bacteria; (2) remarkable peroxidase-like activity for catalyzing the TMB chromogenic reaction, serving as the basis for colorimetric detection of *C. sakazakii*; (3) specific recognition capability towards *C. sakazakii*, thereby enhancing detection specificity. Unfortunately, the practical application of Fe_3_O_4_@EspYZU13 still requires significant advancements. In summary, these distinctive features of Fe_3_O_4_@EspYZU13 ensure its potential application in detecting *C. sakazakii* in powdered infant formula.

## Figures and Tables

**Table 1:** Determination of *C. sakazakii* in spiked PIF samples using the Fe_3_O_4_@EspYZU13 + H_2_O_2_ + TMB reaction system.

Sample	Spiked	Detected	RSD	Recovery	PC
	(CFU mL^−1^)	(CFU mL^−1^)	(%)	(%)	(CFU mL^−1^)
PIF 1	6.4 × 10^3^	6.3 × 10^3^	0.28	98.44	6.2 × 10^3^
	6.4 × 10^5^	6.2 × 10^4^	4.21	96.76	6.4 × 10^5^
PIF 2	6.4 × 10^3^	6.6 × 10^3^	3.15	103.13	6.5 × 10^3^
	6.4 × 10^5^	6.5 × 10^5^	1.97	101.56	6.3 × 10^5^

## Data Availability

The data presented in this study are available on request from the corresponding author.

## Supplementary Materials

The following supporting information can be downloaded at https://www.mdpi.com/article/10.3390/foods13233788/s1: Figure S1. Reaction time-dependent absorbance (652 nm) change of four reaction systems; Figure S2. Effect of buffer pH on the peroxidase-like activity of Fe_3_O_4_@EspYZU13; Figure S3. Storage stability of Fe_3_O_4_@EspYZU13; Figure S4. Steady-state kinetics of Fe_3_O_4_@EspYZU13 using H_2_O_2_ as substrate; Figure S5. Steady-state kinetics of EspYZU13@Fe_3_O_4_ using TMB as substrate; Figure S6. Absorbance of Fe_3_O_4_@EspYZU13 + H_2_O_2_ + TMB reaction system with dead *C. sakazakii*; Table S1. Comparison of kinetic parameters between Fe_3_O_4_@EspYZU13 and the previously reported peroxidase-like nanozymes; Table S2. Comparison of detection performance of our proposed Fe_3_O_4_@EspYZU13 + H_2_O_2_ + TMB reaction system with other previously reported methods.
