# Supplementary material for: Integrating Bacteriophage onto the Magnetic Nanozyme for Effective Enrichment and Colorimetric Detection of Cronobacter sakazakii in Powdered Infant Formula
Source: Foods. 2024 Nov 25;13(23):3788. doi: 10.3390/foods13233788 (PMC11639770; doi:10.3390/foods13233788)
Supplement: Supplementary file 1 [file foods-13-03788-s001.zip › foods-3329836-supplementary.pdf]

Supplementary Materials for

Integrating bacteriophage onto the magnetic nanozyme for effective enrichment and colorimetric detection of *Cronobacter sakazakii* in powdered infant formula

Xuechao Xu[a, 1], Yuansong Zhang[a, 1], Lu Gao[a], Juanli Yang[a], and Zhenquan Yang[a, b, *]

[a] School of Food Science and Engineering, Yangzhou University, Yangzhou, 225127, Jiangsu, China

[b] Yangzhou Engineering Research Center of Food Intelligent Packaging and Preservation Technology, Yangzhou University, Yangzhou, 225127, Jiangsu, China

*Corresponding author

[1] The authors have equal contribution to this work.

E-mail addresses: yangzq@yzu.edu.cn

S1. Chemicals and reagents

Phosphotungstic acid, NH_4OH, ascorbic acid, $CHCl_3$, 4-morpholineethanesulfonic acid (MES), $MgSO_4 \cdot 7H_2O$, N-hydroxy succinimide (NHS), ethanol, sodium acetate (NaAc), and acetic acid (HAc) were obtained from Sinopharm Chemical Reagent Co., Ltd. in China. $FeCl_3 \cdot 6H_2O$, $FeCl_3 \cdot 6H_2O$, 5, 5-dimethyl-1-pyrroline N-oxide (DMPO) and TMB were procured from Shanghai Aladdin Biochemical Technology Co., Ltd in China. 1-(3-Dimethylaminopropyl)-3-ethylcarbodiimide hydrochloride (EDC·HCl) was purchased from Shanghai McLean Co., Ltd in China. Nutrient broth (NB), Luria-Bertani (LB), agar powder medium, and *C. sakazakii* chromogenic medium (DFI agar) were procured from Huankai Microbial Sci. & Tec. Co., Ltd. in China. SYBR-Gold nucleic acid gel stain and 4', 6-diamidino-2-phenylindole (DAPI) were purchased from Thermo Fisher Technology Co., Ltd. in China. All chemicals and reagents were used as received without further purification unless otherwise stated. Deionized water was utilized throughout the study.

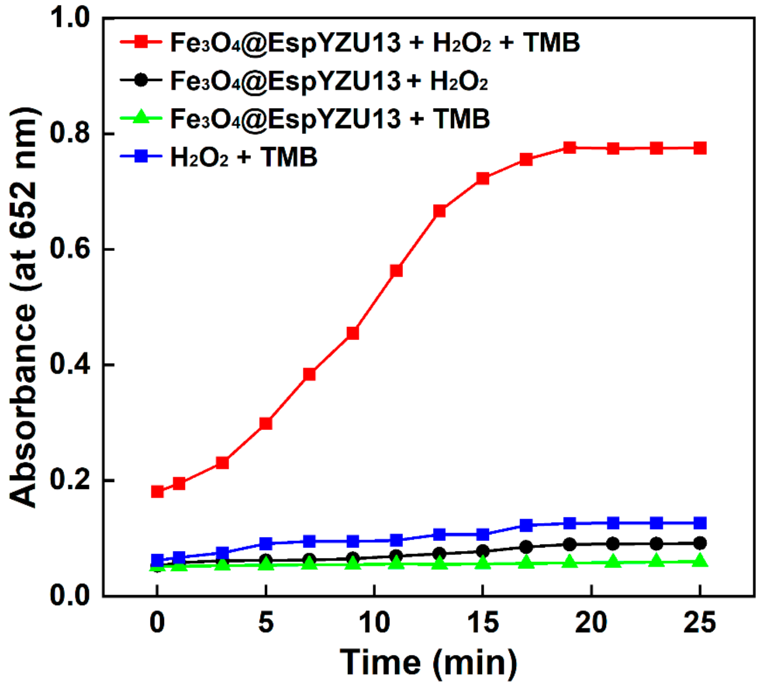

Figure S1. Reaction time-dependent absorbance (652 nm) change of four reaction systems.

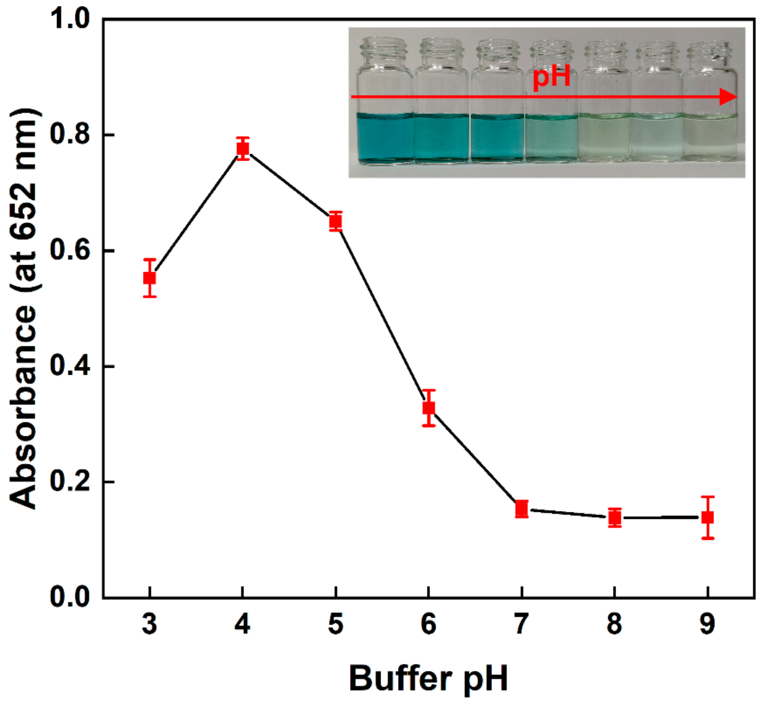

Figure S2. Effect of buffer pH on the peroxidase-like activity of Fe_3O_4@EspYZU13.

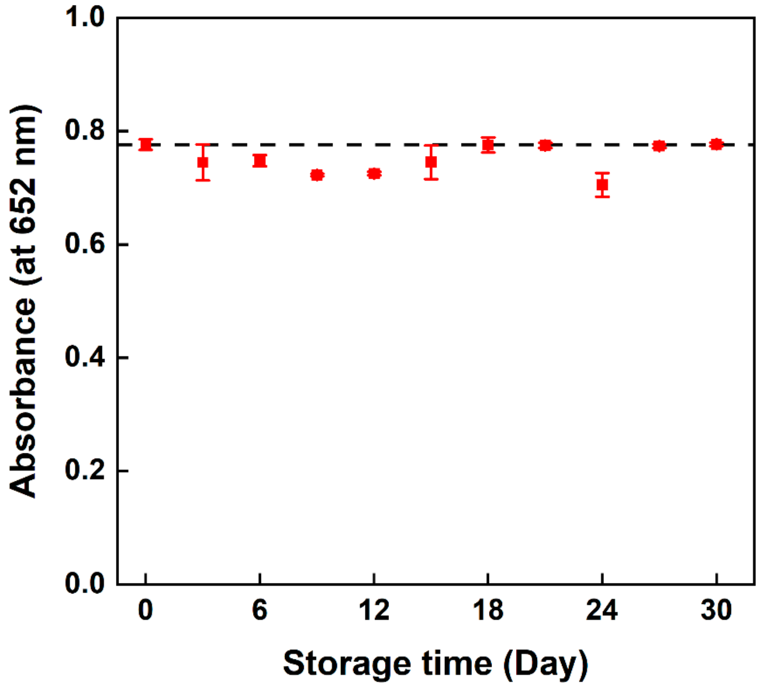

Figure S3. Storage stability of Fe_3O_4@EspYZU13.

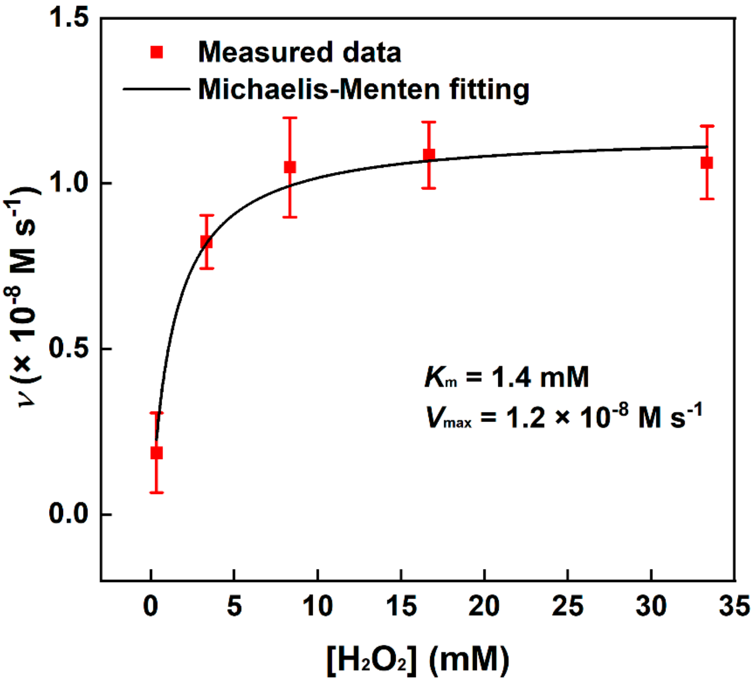

Figure S4. Steady-state kinetics of Fe_3O_4@EspYZU13 using H_2O_2 as substrate.

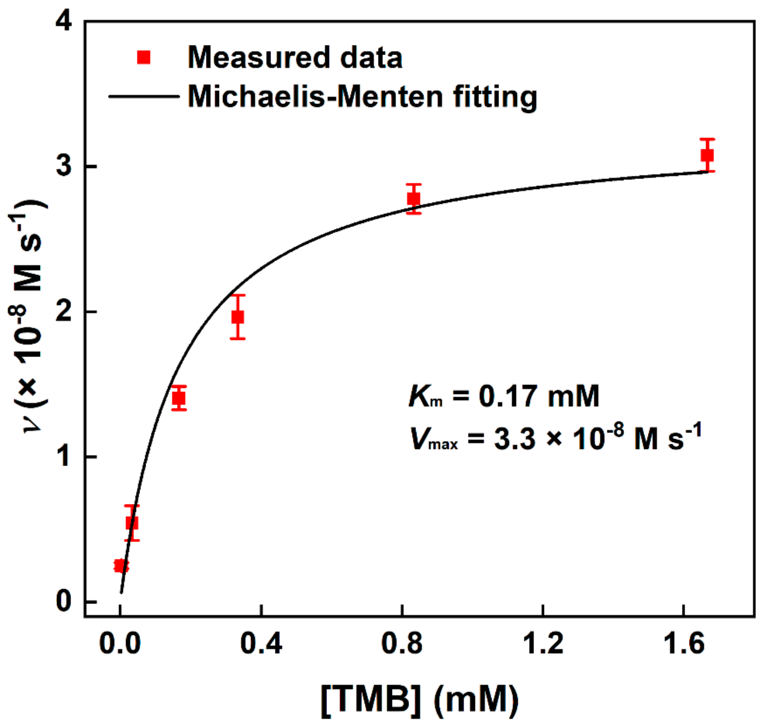

Figure S5. Steady-state kinetics of EspYZU13@Fe_3O_4 using TMB as substrate.

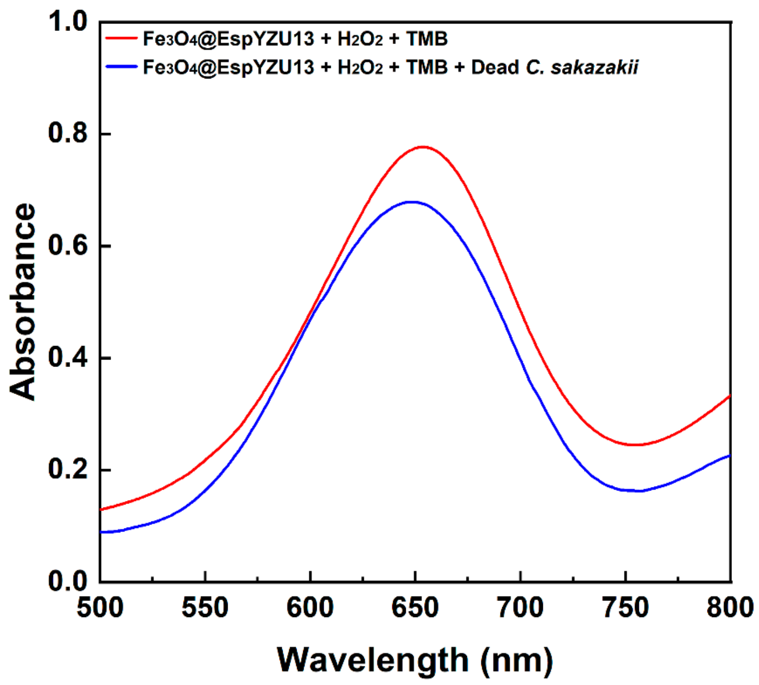

Figure S6. Absorbance of Fe_3O_4@EspYZU13 + H_2O_2 + TMB reaction system with dead *C. sakazakii*.

Table S1. Comparison of kinetic parameters between Fe_3O_4@EspYZU13 and the previously reported peroxidase-like nanozymes.

Nanozyme	K_m (mM)		V_{max} ($\times 10^{-8}$ M s^{-1})		Ref.
	H_2O_2	TMB	H_2O_2	TMB	
SapYZU15@Fe_3O_4	11.5	6.0	2.2	16.3	[1]
EspYZU15@Pd	8.3	0.6	1.9	9.1	[2]
Fe_3O_4@EspYZU13	1.4	0.17	1.2	3.3	This work

Table S2. Comparison of detection performance of our proposed Fe_3O_4@EspYZU13 + H_2O_2 + TMB reaction system with other previously reported methods.

Material	Method	Detection range (CFU mL^{-1})	LOD (CFU mL^{-1})	Ref.
GO/Au	Electrochemistry	$2 \times 10^2 \sim 2 \times 10^7$	20	[3]
mAb-Au-PATP	SERS	$10^2 \sim 10^7$	201	[4]
DNAzyme	Electrochemistry	$3.84 \times 10^4 \sim 2.4 \times 10^7$	501	[5]
EspYZU15@Pd	Colorimetry	$3.2 \times 10^2 \sim 3.2 \times 10^7$	36	[6]
Fe_3O_4@EspYZU13	Colorimetry	$3.2 \times 10^1 \sim 3.2 \times 10^7$	26	This work

Reference

1. Zhou, W.; Wen, H.; Hao, G.; Zhang, Y.-S.; Yang, J.; Gao, L.; Zhu, G.; Yang, Z.-q.; Xu, X. Surface engineering of magnetic peroxidase mimic using bacteriophage for high-sensitivity/specificity colorimetric determination of *Staphylococcus aureus* in food. *Food Chemistry* ***2023****, 42*6, 136611.
2. Zhang, Y.-s.; Xu, X.; Yang, J.; Tan, M.; Zhou, W.; Gao, L.; Yang, Z.-q. Directional immobilization of phage on the palladium-based nanozyme for colorimetric detection of *Cronobacter sakazakii* in powdered infant formula. *LWT* ***2023****, 186*, 115260.
3. Shukla, S.; Haldorai, Y.; Bajpai, V.K.; Rengaraj, A.; Hwang, S.K.; Song, X.; Kim, M.; Huh, Y.S.; Han, Y.-K. Electrochemical coupled immunosensing platform based on graphene oxide/gold nanocomposite for sensitive detection of *Cronobacter sakazakii* in powdered infant formula. *Biosensors and Bioelectronics* ***2018****, 109*, 139-149.
4. Gao, S.; Wu, J.; Wang, H.; Hu, S.; Meng, L. Highly sensitive detection of *Cronobacter sakazakii* based on immunochromatography coupled with surface-enhanced Raman scattering. *Journal of Dairy Science* ***2021****, 104*, 2748-2757.
5. Yuan, Y.; Wu, X.; Liu, Z.; Ning, Q.; Fu, L.; Wu, S. A signal cascade amplification strategy based on RT-PCR triggering of a G-quadruplex DNAzyme for a novel electrochemical detection of viable *Cronobacter sakazakii*. *Analyst* ***2020****, 145*, 4477-4483.
6. Lv, X.; Gu, X.; Wang, L.; He, X.; He, C.; Zhang, J.; Zhao, L. Rapid and absolute quantification of VBNC *Cronobacter sakazakii* by PMAxx combined with single intact cell droplet digital PCR in infant foods. *LWT* ***2021****, 145*, 111388.
